# "Harnessing genomics to improve health in Africa" – an executive course to support genomics policy

**DOI:** 10.1186/1478-4505-3-2

**Published:** 2005-01-24

**Authors:** Alyna C Smith, John Mugabe, Peter A Singer, Abdallah S Daar

**Affiliations:** 1 Joint Centre for Bioethics, University of Toronto, 88 College Street, Toronto, ON, M5G 1L4, Canada; 2 Science and Technology Forum, NEPAD, Pretoria, South Africa; 3 Department of Medicine, University of Toronto, Canada; 4 McLaughlin Centre for Molecular Medicine; Department of Public Health Sciences and Department of Surgery, University of Toronto, Canada

## Abstract

**Background:**

Africa in the twenty-first century is faced with a heavy burden of disease, combined with ill-equipped medical systems and underdeveloped technological capacity. A major challenge for the international community is to bring scientific and technological advances like genomics to bear on the health priorities of poorer countries. The New Partnership for Africa's Development has identified science and technology as a key platform for Africa's renewal. Recognizing the timeliness of this issue, the African Centre for Technology Studies and the University of Toronto Joint Centre for Bioethics co-organized a course on Genomics and Public Health Policy in Nairobi, Kenya, the first of a series of similar courses to take place in the developing world. This article presents the findings and recommendations that emerged from this process, recommendations which suggest that a regional approach to developing sound science and technology policies is the key to harnessing genome-related biotechnology to improve health and contribute to human development in Africa.

**Methods:**

The objectives of the course were to familiarize participants with the current status and implications of genomics for health in Africa; to provide frameworks for analyzing and debating the policy and ethical questions; and to begin developing a network across different sectors by sharing perspectives and building relationships. To achieve these goals the course brought together a diverse group of stakeholders from academic research centres, the media, non-governmental, voluntary and legal organizations to stimulate multi-sectoral debate around issues of policy. Topics included scientific advances in genomics innovation systems and business models, international regulatory frameworks, as well as ethical and legal issues.

**Results:**

Seven main recommendations emerged: establish a network for sustained dialogue among participants; identify champions among politicians; use the New Plan for African Development (NEPAD) as entry point onto political agenda; commission an African capacity survey in genomics-related R&D to determine areas of strength; undertake a detailed study of R&D models with demonstrated success in the developing world, i.e. China, India, Cuba, Brazil; establish seven regional research centres of excellence; and, create sustainable financing mechanisms. A concrete outcome of this intensive five-day course was the establishment of the African Genome Policy Forum, a multi-stakeholder forum to foster further discussion on policy.

**Conclusion:**

With African leaders engaged in the New Partnership for Africa's Development, science and technology is well poised to play a valuable role in Africa's renewal, by contributing to economic development and to improved health. Africa's first course on Genomics and Public Health Policy aspired to contribute to the effort to bring this issue to the forefront of the policy debate, focusing on genomics through the lens of public health. The process that has led to this course has served as a model for three subsequent courses (in India, Venezuela and Oman), and the establishment of similar regional networks on genomics and policy, which could form the basis for inter-regional dialogue in the future.

## Background

Inequities in global health continue to be among the major challenges facing the international community [1]. Despite tremendous advances in medicine, the benefits of science and technology have yet to make a major impact on the health and quality of life of majority of the world's population. Recognizing its fundamental role as engine for development, the New Partnership for Africa's Development (NEPAD) has identified science and technology as a key platform for Africa's renewal [2]. A major challenge for Africa, and for the entire international community, is to bring scientific and technological advances to bear on the health priorities of poorer countries [3,4].

Africa in the twenty-first century is faced with a heavy burden of disease, combined with ill-equipped medical systems and underdeveloped technological capacity [5]. The crippling poverty in many countries in the continent contributes to the disease burden, and hampers countries' ability to address the problem adequately [6]. While Africa's response to its health challenges has varied considerably across the continent, with governments traditionally placing less emphasis on developing S&T than other sectors [7], there has been ongoing R&D activity in genomics and related fields of technology over the past several years in various parts of the region. The African Medical Research Foundation (AMREF), Africa's largest indigenous health charity, has for nearly half a century made an important contribution to addressing health challenges in Africa through partnerships with local communities, governments and donors [8]. A number of centres of excellence have emerged across the continent in recent decades, including the International Centre of Insect Physiology and Ecology (ICIPE) in Nairobi where important work has been done to uncover the role of insects in the transmission of infection , and the Institute for Molecular and Cell Biology-Africa (IMCB-A), founded in 1999 to study the molecular mechanisms of tropical infections. A further example is the new Biosciences Facility for Eastern and Central Africa that was recently launched as part of a NEPAD initiative [9]. NEPAD, which has been adopted by the United Nations General Assembly as Africa's development framework, has called "for the establishment of regional platforms with concrete actions to build and strengthen Africa's competence to harness and use new technologies for human development" [2]. Its strategy acknowledges that Africa will have to overcome considerable challenges, including creating adequate regulatory and biosafety frameworks, building scientific capacity, and developing integrated systems of innovation.

In March 2002, the African Centre for Technology Studies (ACTS) and the University of Toronto Joint Centre for Bioethics (JCB) co-organized an intensive five-day Course on Genomics and Public Health Policy in Nairobi, Kenya, bringing together scientists, policy makers, journalists, lawyers and NGOs from ten African countries to discuss, collectively, the question of "How best to harness genomics to improve health in Africa?" This course was sponsored by Genome Canada, the International Development Research Centre, and the African Centre for Technology Studies, through the Norwegian Agency for Development Co-operation. The primary goal of the course was to familiarize participants with the potential of genomics and related biotechnologies to address health needs in Africa. This article presents the findings and recommendations that emerged from this process, and suggests how such courses might be more broadly employed as a method for bringing together opinion leaders to share ideas and work collectively to develop practical policy solutions.

## Methods

The programme was planned collaboratively by the African Centre for Technology Studies and the Joint Centre for Bioethics. The basic layout of the sessions and their topics was modelled on a prior course held in Toronto, Canada in May 2002. The programme was organized in line with the objectives outlined in Table 1. Course participants as well as session leaders were identified on the basis of recommendations from recognized experts in the region and through literature searches. Many session leaders were local experts, well placed to contextualize the "new science" of genomics within the frame of concerns and realities particular to Africa. Care was taken to select participants representing a range of interests and backgrounds, including individuals from science, economics, law, government, the press, and non-governmental organizations. Such diversity was sought in recognition of the importance of "cross-pollination" on a multifaceted topic like genomics, and consequently the need for multiple actors to be part of the building of policy, as well as mediating the dialogue between policymakers and the public. In total, 30 participants attended; the countries and the institutions they represent are listed in Table 2. Despite concerted efforts to draw a balanced group, the participant list reveals a markedly high proportion of academics, and indeed no representatives from industry. Moreover, only three of the participants are women. The organizers covered all costs for attending the course (transportation, hotel accommodation, and meals), in order that inability to pay not be an inhibiting factor for those who wished to participate.

**Table 1:** Objectives of the course

• To familiarize participants with the current status and implications of genomics and biotechnology for health in India, and to provide information relevant to public policy
• To provide frameworks for analyzing and debating the policy issues and related ethical questions, and to help understand, anticipate and possibly influence the legal and regulatory frameworks which will operate, both nationally and internationally
• To begin developing an opinion leaders network across different sectors (industry, academic, government, and voluntary organizations) by sharing perspectives and building relationships

**Table 2:** Countries and Institutions Represented

African Centre for Technology Studies, Kenya
African Malaria Vaccine Testing Network (AMVTN), Tanzania
African Medical and Research Foundation, Kenya
Centre for the Development of People (CEDEP), Uganda
Chemistry Department, University of Zambia, Zambia
Department of Biochemistry, University of Khartoum, Sudan
Department of Epidemiology of Parasitic Disease, National School of Medicine and Pharmacy, Mali
Department of Obstetrics and Gynecology, Assiut University, Egypt
Department of Pathology, Makarere University, Uganda
Department of Virology, University of Ibadan, Nigeria
Division of Human Genetics, Faculty of Health Sciences, University of Cape Town, South Africa
Dysmorphology and Alcohol Pharmacokinetics in Fetal Alcohol Syndrome, South Africa
Federal Ministry of Science and Technology, Nigeria
Inter-Region Economic Network (IREN), Kenya
Journalist Against AIDS (JAAIDS), Nigeria
Lawyer, Kenya
Maternal, Child and Women's Health, Dpt. of Health, Western Cape Province, South Africa
Molecular Biology Research Facility, Nelson R Mandela School of Medicine, South Africa
National Council for Science and Technology, Kenya
National Health Laboratory Service and Division of Human Genetics, University of Witwatersrand, South Africa
School of Public Health, University of Ghana, Ghana
Science and Development News, and BiotekAfrika, Kenya
Science Secretary, Uganda Council for Science and Technology, Uganda
The People Newspaper, Kenya

Because of the diversity of the participants, no background in science was presupposed. The sessions were organized so that participants were first introduced to the subject of the "new science" of genomics, and were then instructed in areas including national innovation systems, business models, intellectual property rights, international conventions and regulatory structures, ethics, and the role of networks in facilitating dialogue, advocacy and policy making. A detailed time-table of the programme is shown in Table 3. Presenters used overhead transparencies or presentation software such as Microsoft Powerpoint. Active participation was encouraged throughout with at least 45 minutes allotted for discussion at the end of each session, on the assumption that each participant brought considerable expertise and valuable practical experience of his or her own. The programme therefore employed a peer-learning environment in which participants could learn from each other, in addition to learning from material presented by instructors. Each participant was provided with a course reader, which included additional background material on session topics; class sessions used a variety of learning methods including lectures, discussions, case analysis, and simulations.

**Table 3:** Agenda for the Course on Genomics and Public Health Policy in Africa.

**Time**	**Day 1**	**Day 2**	**Day 3**	**Day 4**	**Day 5**
9.00–10.30	Introduction *Prof Abdallah Daar, Dr John Mugabe* New Science I : Introduction *Dr Stephen Scherer*	Internet-based Leader Networking: Exercise *Prof Joseph D'Cruz*	Intellectual Property Rights I *Dr Patricia Kameri-Mbote*	Ethics I *Dr Peter Singer*	Group Presentations

11.00–12.30	New Science II *Dr Stephen Scherer*	National Innovation Systems *Prof Norman Clark*	Intellectual Property Rights II *Dr Patricia Kameri-Mbote*	Ethics II *Prof Abdallah Daar*	Group Presentations Continued

1.30–3.00	New Science III *Prof Onesmo ole-MoiYoi*	Business Models *Prof Joseph D'Cruz*	Internet-based Leader Networking: Results *Prof Joseph D'Cruz*	Science & Innovation Policy in International Conventions *Dr John Mugabe*	

3.30–5.00	Genomics and Global Health *Dr Peter Singer*	*Group Work*	*Group Work*	*Group Work*	

Early in the course, participants were divided into small Study Teams consisting of persons with diverse backgrounds, in order to maximize complementary skills. These Study Teams were an integral part of the learning process of the programme. Sessions were intended primarily to provide input for participant Study Teams, which assembled several times during the week. Their primary task was to draw upon the course material and their own experiences to propose recommendations for policy relating to genomics and biotechnology in Africa. Presentations were made on the last day of the course, and the final sessions focused on how to take forward the ideas and proposals generated during the course.

This was the first course of its kind in Africa, as well as the first of a series of planned courses on genomics policy to be held in developing countries; evaluation was therefore a key component of the programme. At the end of each day, participants were given a questionnaire to complete, in which they had an opportunity to evaluate the day's sessions. At the end of the course, participants were asked to complete a more detailed questionnaire, asking for their feedback on the overall aims and organization of the course.

## Results

The course opened with an Introduction, where Prof. Abdallah Daar and Dr John Mugabe welcomed the participants, explained the course's objectives, and then invited each of the participants to introduce him- or herself to the rest of the group. The opening session was led by Dr Stephen Scherer, and was intended to provide a comprehensive overview of the science of genomics and its relevance to health. Several of the participants had a limited scientific background; the presentation therefore include very basic descriptions of the science involved, as well as images and a brief video, and gradually progressed to a discussion of its applications in health research and medicine, both now and in the future. This session was followed by an introduction by Prof. Onesmo ole-MoiYoi, a pioneering Kenyan scientist, to advances in genomics and molecular biology within the African context – including cutting-edge research at his institute and others on the continent, as well as the broader relevance of genomics and molecular approaches to the health of Africa's people, animals and the environment. The first day closed with a session led by Dr Peter Singer who described a five-point strategy to systematically capture the benefits of genomics for the health of citizens in developing countries, through research, capacity-strengthening, consensus-building, public engagement, and an investment fund. Examples of ongoing work by the University of Toronto's Canadian Program on Genomics and Global Health in these areas were discussed, including the results of its 2002 study to identify the most promising biotechnologies to improve health in developing countries [13].

Prof. Joseph D'Cruz opened the second day with a discussion introducing participants to new approaches to forming and expressing opinions about emerging issues using the internet. Leaders in any area are required to develop their own views about new developments in their fields, and the process of forming these views is facilitated by peer discussions. Though traditionally these processes have taken place face-to-face, the internet offers an alternative medium that allows individuals to interact with their peers in other locations at a time and pace suited to each individual's commitments, without forcing the group to reach early consensus. Prof. Norman Clark followed with a session aimed at introducing participants to the concept of 'National Systems of Innovation', a conceptual framework for analysing country-specific factors that influence innovation across sectors. Innovation is understood as processes of generating new ideas, products and production processes, as well as to processes of institutional change and development. Such frameworks can be useful in identifying and analyzing key factors affecting African countries' ability to engage effectively in biotechnology and genomics for human development. The last session of the day focused on the business life cycle of a genomic product, tracing its development from the laboratory bench to a patented invention that is exploited commercially. The session addressed the strategic issues and choices that firms face at each point in this life cycle, and used a case-study based approach to frame the issues. The last one-and-a-half-hour session of the day was devoted to group work among members of Study Teams, whose members were selected to bring diverse views and experiences to bear on their deliberations.

The third day of the course was devoted primarily to the issue of intellectual property rights (IPRs). The two sessions on IPRs were led by Dr Patricia Kameri-Mbote, Kenyan lawyer and scholar. During the first of these, Dr Kameri-Mbote explained the nature and different kinds of IPR protection, and explored how these impact on biotechnology development and technology transfer. She also considered the relationship between IP protection and public health in developing countries, using specific cases that have arisen under the World Trade Organization's Agreement on Trade Related Aspects of Intellectual Property Rights (TRIPS). Positions held by different countries and scholars on IP and biotechnology transfer in health were examined, and international, regional and national intellectual property regimes were reviewed. The second session focused on the link between IP, public health and transfer of biotechnology, in addition to the ethical, social and policy implications of the "Doha Declaration" on health by WTO ministers intellectual property rights in the area of health under TRIPS. At the end of the third and fourth days, participants again met for 1.5 hours in their Study Teams to prepare their proposals.

Day four of the course had a heavy focus on ethical dimensions of emerging technologies like genomics. The first session provided an overview of ethical issues related to genomics and public health policy. Prof. Abdallah Daar led this and the second session on ethics. He described the World Health Organization's draft Guiding Principles on Medical Genetics and Biotechnology document, which he co-authored and which provides a broad overview of the ethical principles in this field. During the second session, Prof. Daar and Dr. Singer led the group through a case involving benefit sharing, and introduced the Human Genome Organization's principles and statement on benefit sharing. Dr. Singer then described an ethical framework and approach to priority-setting for genomics technologies in health care institutions. The last hour of this session was devoted to providing a forum for participants to share their expertise and experiences in areas related to policy. The final session of the day was led by Dr John Mugabe, then-Director of the African Centre for Technology Studies in Nairobi, Kenya. This session introduced participants to international conventions and protocols that emerged out of the United Nations Conference on Environment and Development (UNCED), and focused on science and innovation issues covered by the Conventions on Biological Diversity and its Cartagena Protocol on Biosafety, and the International Treaty on Plant Genetic Resources for Food and Agriculture. Specific lessons were drawn for international rule-making for health equity, and emphasis was given to biotechnology, risks assessment, technology transfer, sharing benefits of global scientific and technological advances, and technical cooperation.

On the last day, each of the four Study Teams presented their proposals, which addressed the overarching question of the course: "How to harness genomics and related biotechnology to improve health in Africa?" Study Teams presented one at a time; after each presentation, there was a period for questions and discussion, and afterward an opportunity to consider all proposals together in light of the host of issues raised during the course of the week. The presentations, though prepared independently by each group, demonstrated a number of common themes that tended to be organized in terms of long-term foundational issues of sustainability, and more concrete short-term issues relating to garnering political involvement. Table 5 enumerates the key recommendations that emerged from these sessions.

**Table 5:** Recommended Action-Steps

Establish a regional network to foster sustained inter-sectoral dialogue
Identify champions among politicians
Use the New Plan for African Development (NEPAD) as entry point onto political agenda
Commission African capacity survey in genomics-related R&D to determine areas of strength
Undertake a detailed study of R&D models with demonstrated success in the developing world
Establish seven regional research centres of excellence
Create sustainable financing mechanisms

## Discussion

The following is a synthesis of the participants' efforts, summarizing and describing key issues that emerged from their presentations and throughout the weeks' deliberations. It includes several concrete action-steps recommended by the participants, which flow from these considerations.

### Creating a Platform for Ongoing Dialogue and Advocacy

The course generated a great deal of enthusiasm and vigorous discussion, and there was consensus among the participants on the need to create a mechanism for capitalizing on this momentum. Course participants and faculty therefore established an e-mail-based network, the *African Genome Policy Forum (AGPF)*, to allow the continued exchange of ideas and the building of consensus on issues related to genomics and public health policy. The group, composed of participants from areas of government, academia, civil society and the media, was created to bring to the table the views of their respective constituencies, and inform their peers of insights gained from the course and through the network. The network may also play an advocacy role in promoting the responsible use of genomics as a tool to improve health and promote development in Africa.

#### *Concrete Action-Step 1*: Establish a regional network to foster sustained inter-sectoral dialogue

On the final day of the course, it was decided that a regional network, the "African Genome Policy Forum", be established comprising all participants and session leaders; it was further agreed that the Joint Centre for Bioethics would set up a web-site, discussion board, and e-mail based platform to facilitate ongoing discussion and inter-sectoral debate on the issues and proposals raised during the course.

### Mobilizing Political Support

The success of any major initiative requires sustained dialogue with politicians. It is important to take the time to address their legitimate concerns, by clarifying the specific relevance of genomics and its applicability within the context of their communities. A point of particular relevance is the link between technologies like genomics and Africa's development, which has been well described in a number of recent reports [*e.g*. [6,10]]. Participants highlighted the importance of taking back to their colleagues in their respective countries and institutions the lessons drawn from the course; those participants in public office agreed to seize opportunities to raise some of issues and proposals of the course when attending relevant forums. In particular, the nascent New Partnership for Africa's Development, adopted in 2001 under the mandate of the Organisation of African Unity, was repeatedly pointed to as an opportunity to bring genomics and its relevance to health in Africa onto the political agenda. Science and technology is among NEPAD's seven priority areas; another is human development, which encompasses health [11]. Genomics provides a clear example of how these two areas – science and technology, and health – come together, and can serve as a model for considering how science and technology and health concerns can be better integrated to address the continent's economic and health needs.

#### *Concrete Action-Step 2*: Identify champions among politicians

The most efficient means of garnering political support is often to go directly to the politicians themselves – those who have been supportive or outspoken of the issues in question – to put the subject before their colleagues. The course itself represented an important step in this direction, as it brought together a spectrum of stakeholders, including academics, civil society, and government officials. The course, and the subsequently established network, therefore furnished an opportunity for direct communication and dialogue among individuals with a shared vision, including policymakers in a position to "champion" the issues and proposals that emerged from the course to their colleagues and others.

#### *Concrete Action-Step 3*: Use the New Plan for African Development (NEPAD) as entry point onto political agenda

NEPAD offers a possible forum to bring the subject of genomics-related biotechnology onto the political agenda, and provides a means of informing African leaders of genomics and its relevance to improving health and development in Africa. In particular, the AGPF recommends the establishment by NEPAD of an 'African Genomics Committee', which would provide a plan for utilizing genomics and other new technologies to enhance health in Africa, advocate for increased investment in S & T, target other relevant stakeholders in individual countries, educate policy makers about the need for a strong R&D base established through partnerships across Africa, and organize steering committees to identify gaps and implement strategies for improvement.

### Prioritizing Needs

Participants agreed on the need to consider emerging technologies like genomics in light of Africa's specific health challenges, and consequently on the importance of prioritizing these and identifying strategic entry points. Infectious (including sexually transmitted) diseases, genetic and other non-communicable disorders, sanitation, nutrition, environmental pollution and loss of biodiversity were all proposed as areas requiring concerted attention, with a special emphasis on the potential for using genomics-related biotechnology to target the three biggest killers in Africa: malaria, HIV/AIDS and tuberculosis. There are already well-known African-led initiatives to apply scientific innovation to combat important health concerns, such as the Multilateral Initiative on Malaria, and the African Malaria Vaccine Testing Network (AMVTN). It will be important to build on existing success stories, and to identify gaps in terms of priority health areas receiving inadequate attention. This will help to focus efforts and to more efficiently channel limited resource, both financial and human. A regional approach, which has since been adopted by NEPAD, was proposed as a promising mechanism for harnessing existing competence to address local needs.

#### *Concrete Action-Step 4*: Commission African capacity survey in genomics-related R&D to determine areas of strength

This survey would identify strategic areas of strength, such as existing centres of excellence, potential areas of improvement, and health priorities receive inadequate attention. It would also serve to identify local and national innovators, and to inform the structuring of Regional Centres of Excellence described below.

### Capacity Building & Public Engagement

For several years, genomics has been linked with a number of high-profile, intensely controversial issues like human cloning and genetically modified organisms. While emerging technologies like genomics raise a number of important ethical and social issues that deserve careful consideration [12], a nuanced message takes account of the possibilities as well as the challenges of new approaches. Often, technological applications can complement existing, well-established health approaches [13]. Scientists, policy-makers, and the media have an important part to play in publicizing science, and pointing out its relevance to Africans in a moderate rather than hyperbolic tone [14]. Local leaders can have an important role to play, not only in reflecting the leading-edge opinions of their different constituencies to policymakers, but also by playing a role in raising awareness within their communities. A more informed public is often a more engaged public, which can effectively advocate for the development of policies that reflect legitimate concerns, while leaving space to explore promising avenues of scientific endeavour.

*Public engagement *was seen to form part of a long-term strategy for capacity building, and raising the overall profile of science and technology in Africa. The discussions reflected a conception of capacity strengthening as intimately linked with quality education – at all levels, and across disciplines. Core to this debate among course participants was the belief that endogenous capacity must be developed in order that Africa can begin to be self-sufficient, and itself become an innovator. Participants identified the following categories as needing attention:

#### Primary, secondary and tertiary education

There is a need to introduce innovative techniques to teach science and technology in the classroom, in order to generate interest and aptitude in the subject matter from an early stage in the educational process. Besides contemporary scientific approaches, indigenous knowledge and its applications to health could also be a relevant component to include in the curriculum.

#### Policymakers

Those in a position to shape policy should be familiarized with codes of ethics pertaining to their field; moreover, they should be educated about how best to capitalize on international frameworks (e.g. WTO's Trade-Related Aspects of Intellectual Property Rights; the UN's Convention on Biological Diversity) in order to ensure that their countries benefit from such arrangements, and are not exploited. Policy makers should develop strategies for negotiating their interests collectively in international forums, when appropriate, given shared needs and values.

#### Media

There is a general need to strengthen capacity in the area of communication, in particular on increasing the level of science literacy among the media. This might include integrating journalism and science programs at the college and university levels. There is a corresponding need to improve the ability of scientists to communicate the relevance of their work to the public, and to policy-makers.

#### ELSI

There is a great need to build capacity in Africa with regard to the ethical, legal and social issues (ELSI) which inevitably accompany the emergence of new technologies. Strategies would in many cases involve sensitizing the public to issues of relevance, such as their rights as patients and participants in research (e.g. informed consent, confidentiality of patient information), encouraging dialogue about the social consequences of introducing new technologies into traditional settings, and putting frameworks in place (e.g. ethics review boards) to ensure that ethical, quality and safety standards before research is undertaken.

### Partnerships

Along with the need to strengthen the R&D base in science and technology, participants of the course identified a related need to increase the emphasis on commercialization – not only as a tool for sparking innovation but also to permit the generation of capital necessary to sustain the industry. An important step in the process of moving toward commercialization is the forming of alliances within countries, between universities and industry, sometimes known as "cross-linking". The fruitfulness of the Africa course, where people from across sectors and sub-regions came together with a common mission, re-enforced the value and the importance of establishing cross-sectoral networks and collaborations. *Networks *provide a means of generating new ideas, pooling the creative energies of individuals, and exchanging advice and expertise around a particular area of focus, in this case genomics and health policy. Such networks could play an advocacy role, combining the voices and the influence of key players from diverse disciplines and sectors, to advance a common aim. *Collaborations*, at the level of institutions – both within and between countries and regions – would facilitate the transfer of both knowledge and technology. During the course, it was pointed out that there is a particular need to encourage linkages between universities and industry to, among other things, facilitate the move from research and development to product generation and commercialization. This could include mechanisms to facilitate relationships between universities undertaking research in biotechnology and local industries. Institutional partnerships and collaborations at all levels, including internationally, can mean the channelling of resources to common areas of focus, and pooling the relative strengths and resources of partner institutions [15]. Such collaborations require very clearly defined roles for partners, and transparency with respect to goals, prioritization of needs, funding, and mechanisms to ensure equitable access to products.

### Creating sustainable financing mechanisms

Ensuring that the benefits of science and technology, including emerging fields like genomics, requires a long-term strategy for sustained investment.

#### *Concrete Action-Step 5*: Design proposals for obtaining sustained investment for both research and development (R&D) in genomics and related biotechnologies to improve health, and the commercialization of the products of R&D

Three models were suggested

*The establishment of an African Science and Technology Fund*, dedicated to supporting research and development in the area of health-related biotechnology, would rely upon the contribution of African governments.

*The establishment of an Investment Fund for genome-related biotechnologies for improving health *would represent an innovative approach to obtaining capital, providing a further incentive for investors to put money into development by creating a fund that provides a return on investment, as well as furnishing funds for advancement. Such a fund might be dedicated to providing capital for the development of mature, or future, health-related technologies.

*Capitalizing on existing funds *allocated for research related to diseases afflicting Africa, such as the WHO's Global Fund to Fight   AIDS, Tuberculosis and Malaria. Genomics and biotechnology represents a powerful set of tools for health improvement, and the World Health Organization through its *Genomics and World Health *(2002) report has raised it as an important issue deserving international attention. It is important to use this positive emphasis to give weight to the case for the relevance of biotechnology to health in developing countries, particularly for policy makers.

### Research and Development (R&D)

With respect to R&D, there are already areas of strength on the continent; it is crucial to identify localized expertise, and to establish linkages with centres elsewhere in the region, as well as abroad, to ensure the transfer of knowledge and of technology, and to facilitate human resource development. Infrastructure must be developed to attract qualified African researchers to remain in or to return to Africa – both to support them, technically, intellectually, and socially and to provide them with similar opportunities for creativity and growth as may be found in other locales. The Biosciences Facility, established in 2003 by NEPAD, takes up this challenge, promoting "scientific excellence by bringing together a critical mass of scientists drawn from national, regional and international institutions in state-of-the-art facilities where they can undertake cutting-edge research to help solve the most important development constraints faced by the poor in Africa" [9]. While the new Biosciences Facility is the first of network of centres of excellent focused primarily on using science to help poor farmers, it may be an appropriate model for like initiatives using a regional approach for targeting health challenges.

#### *Concrete Action-Step 6*: Undertake a detailed study of R&D models with demonstrated success in the developing world, i.e. China, India, Cuba, Brazil

Developing countries in various parts of the world have proven that they too can have strong technology sectors, and make important contributions in terms of science and innovation. Their successes represent an opportunity to bring to the attention of politicians that there are countries succeeding in genomics. A detailed study of these models can provide important insights into how Africa can capitalize on the promise of genomics and biotechnology, particularly as it relates to health. In 2003, the Joint Centre for Bioethics completed a qualitative study of R&D in biotechnology in South Africa; similar studies are underway in Cuba, Egypt and China. Research of this kind could feed into more systematic efforts in the region to better understand how some developing countries, including those in Africa, have managed to develop S&T research and manufacturing capacity in the health sector.

#### *Concrete Action-Step 7*: Establish Seven Regional Research Centres of Excellence

The proposed centres would be distributed across Northern, Southern, Eastern, Western and Central African sub-regions. Each centre would have its own area of focus, in terms of targeted health problems, depending on regional expertise. The Centres would not be the sole preserve of each region, but would in fact use the strengths and specializations of each region to achieve the goal of harnessing genomics to improve health in *Africa*. These regional centres of excellence need not preclude the existence of national centres of excellence. The Biosciences Facility is modelled on such an approach.

## Conclusion

### Analysis

The course on Genomics and Public Health Policy in Africa was carefully designed, with inputs from both its Canadian and African co-organizers, to have a programme and participant profile reflecting the inter-disciplinarity of the issue being considered. Genomics cuts across S&T, environmental, development, industrial, education and health policy and generates important ethical, legal and social issues. It therefore requires a genuinely participatory and multi-stakeholder approach, as well as frank discussions about both the potential promise and perils of a relatively new science.

The strength of the course, as reflected in the evaluations submitted by participants, was the rare opportunity for discussion and networking among opinion leaders from different sectors. Both during and between sessions, participants exchanged perspectives and experiences with others from different regions of the content, and from different disciplines. Senior political officials, journalists, academics, and civil society representatives worked together in Study Teams to create proposals. Discussions were lively and open, with broad participation from those in attendance. However, a weakness of the course was the absence of industry representatives, who would certainly have contributed an important and valuable point of view. The small number of women participants was also a notable disadvantage. Later courses modelled on the Nairobi offering (i.e. those in Latin America, the Eastern Mediterranean, and India) had greater success in drawing participants from industry and obtaining a better gender balance. Notably, however, the recommendations that emerged from these courses, while reflecting differences due to regional priorities and context, did not vary considerably despite the broader contribution, particularly from the private sector [20].

A major outcome of the Nairobi course, and one which had strong support from participants, was the creation of a virtual network to facilitate ongoing interaction and discussion. Within two weeks of its completion, a website was created for the course , as well as a web-based discussion board. While there was some initial activity on the discussion board, this eventually subsided, and was soon evident that this approach had failed. In an effort to revive the momentum and to solicit ideas from AGPF members about how to best move forward with the network, a short survey was sent to members asking what their needs were, both in terms of the network as well as in terms of the technical facilities at their disposal. The response rate was extremely low; however, those who provided feedback confirmed what the participation level suggested: namely, that information technology facilities in Africa are such that very few individuals, outside of some well-equipped academic or private institutions, have regular access to the internet. The web-based discussion board was, therefore, in practice a highly unsustainable option for the majority of participants. The point was also raised that it was not enough to be connected electronically; there was also a need to share a more tangible goal or project, and to have a more visible leader from within the group, to galvanize efforts and motivate continued interaction. One respondent explained that finding the time to contribute to such networks is extraordinarily difficult for many Africans, who often "wear many hats". As a result, a general interest was insufficient to justify diverting time from other tasks; a concrete, realizable goal was essential for engaging individuals who already feel over-stretched. As a consequence of these inputs, an email-based forum was established, since most AGPF members have better access to email than to the internet, and a moderator was temporarily appointed over the group. Activity on the forum improved and continues today, more than two years later, though interventions are irregular and generally extend to the sharing of information or material of interest, rather than discussions about issues.

The India course on Genomics and Public Health Policy was held in January 2003, less than one year after the inaugural Nairobi effort. Based on feedback from the previous course, the questionnaire requesting feedback about participants' technical and substantive needs in relation to the creation of a network was distributed during the course, to permit the creation of a network that was much more responsive to the needs of the participants. Moderators from among the participants were nominated before the course' end and their roles clarified, to facilitate the sustainability and autonomy of the network.

Later in 2003, two further courses were held in Oman and in Venezuela, both of which added a further element demonstrating the learning from the first two courses. On both occasions, the Joint Centre for Bioethics collaborated with the Regional Offices of the World Health Organization; in the first instance, with the Eastern Mediterranean office (EMRO) and in the second, with the Pan-American Health Organization (PAHO). This collaboration ensured that the recommendations of each course had an institutional structure through which they could be channelled, to reach the ear of decision-makers. EMRO and PAHO have extensive links with ministries of health within their regions, as well as with representatives from civil society and industry. This provided an opportunity for the results of the course to have a much wider impact. By contract, the impact of the Nairobi course is very much linked to the efforts of individual participants to engage with their constituencies and with the NEPAD initiative, of which one of their members is now a senior actor. The Forum developed following the Nairobi course has not provided a framework to drive action the way it was initially intended; however, it continues to provide a portal for information-sharing and dialogue.

### Final Remarks

The executive course on Genomics and Public Health Policy in Africa was the first of its kind to be held on the continent. The response of participants indicated a tremendous enthusiasm for and interest in discussing the emerging technology of genomics and its applications for addressing the health woes of Africans. The sessions covered a spectrum of topics, from basic science, to ethics, business models and international frameworks – exemplifying the range of intersecting issues relevant to informed discussions about genomics and related policy. The course also was a demonstration of the fruitfulness of a multi-stakeholder approach. An important aim of the course was to encourage network-building and the development of meaningful interactions, as a foundation for sustained dialogue among opinion leaders. Participants were encouraged to develop independent proposals in a collaborative environment, rather than to be passive recipients of "expertise" from the session leaders. The result was a series of concrete proposals for action, and the establishment of an e-network to provide a forum for ongoing communication, discussion and elaboration of the issues and proposals raised during the course. Several participants agreed to raise the proposals and themes articulated to their colleagues; the course also generated some publicity, as journalists invited to attend and to participate actively in the meeting reported on the key issues in various media [[16,17]; see also [18]].

Since the completion of this course, three more offerings have taken place, one in India in collaboration with the Indian Council for Medical Research (ICMR) in January 2003, another in Oman in August 2003, and a third in Venezuela in 2004. A fourth course is being planned for a venue in South-east Asia. The Nairobi offering demonstrated clearly the receptiveness of African researchers and policy makers to such an initiative, and captured the vision of a cross-section of stakeholders around how to ensure that the new wave of scientific promise does not pass them by, or crush them in its wake, but instead is harnessed for better health and to further economic development in their region [19]. The courses in India and Oman similarly gave rise to regional e-networks [20], which may eventually be connected to form an inter-regional forum for dialogue to form a basis for the sharing of experiences and expertise *across *regions in the developing world. Each of the three executive course held to-date has addressed similar themes in relation to genomics and health; but each has also been adapted to the particular context and interests of the host country or region. This has partly been achieved through active collaboration between the Joint Centre for Bioethics and the host institutions. The electronic networks provide a means of generating a long-term impact, driven by participants who are empowered, in their particular capacities, to take forward the ideas shared and the proposals developed through their interaction. The Nairobi course also highlighted the importance of being proactive in soliciting suggestions from participants about creative means of virtual networking that realistically address the poor information technology infrastructure in most parts of Africa. It also was instructive in demonstrating that a network is not itself self-sustaining; it must be driven by a clear, shared vision among participants, and possibly even a concrete and realizable project. Moreover, ideally a moderator from within the group should take leadership in feeding the forum, and motivating ongoing participation.

The New Partnership for Africa's Development (NEPAD) has made science and technology (including genomics and biotechnology) a key platform in its plan for economic renewal [2,9]. Indeed, the recommendations outlined above overlap considerably with those described in a recent document detailing the resolutions of the first science and technology workshop of NEPAD, held in February 2003 [2]. The recent establishment of the African Biosciences Facility as a centre of scientific and technological excellence in the region, is further evidence that the recommendations articulated by the AGPF reflect a more widely shared vision. There is a growing recognition in Africa, and internationally, of the role that genomics and biotechnology can play, not only in alleviating health scourges of the poor, but also in addressing some of their economic concerns. With appropriate emphasis on its health needs, incentives for meaningful partnerships, sound regulatory structures, innovation and foresight, Africa could be in a position to benefit from genomics and related fields of biotechnology. The Course and Genomics and Health Policy in Africa had as its overarching goal that of bringing together a vibrant cross-section of individuals to foster dialogue around this timely issue. The African Genome Policy Forum works to build on this foundation, to sustain the momentum of the course, and to fulfill some of the participants' proposed goals. Perhaps most significantly, this series of courses represents a practical and effective mechanism for drawing together a variety of actors to address an issue of recognized import, which deserves a truly inter-disciplinary approach. Moreover, it is an initiative that generates important debate, but which is ultimately focused around generating *concrete proposals *to inform policymaking.

## Competing interests

The author(s) declare that they have no competing interests.

## Authors' contributions

All authors participated in and contributed to the course. ACS drafted the manuscript. PAS and ASD conceived of the course, refined the manuscript for critical content and approved final version; and with JM, participated the course design and its coordination. AGFP members provided intellectual input, through their lively discussions and proposals during the Course on Genomics & Public Health Policy in Africa, held 4–8 March 2002.

## Funding

The Canadian Program from Genomics and Global Health is funded by several sources listed at . This course was funded primarily by Genome Canada and the International Development Research Centre (Canada). PAS holds a Distinguished Investigator Award from the Canadian Institutes for Health Research. ASD is supported by the McLaughlin Centre for Molecular Medicine. The African Centre for Technology Studies, which hosted the course, was supported by the Norwegian Agency for Development Co-operation.

**Table 4:** Reading materials.

1	Scherer, S.W. 2001. The Human Genome Project. Isuma: Canadian Journal of Policy Research Vol. 2, No. 3, 11–19.
2	OWENS, K., KING, M-C. 1999, Genomic views on human history. Science 286, 451–455.
3	ROSES, A.D. 2000, Pharmacogenetics and the practice of medicine. Nature 405, 857–865.
4	Nature, Human Genome Volume, Vol. 409, Feb. 2001.
5	Science, Human Genome Volume, Vol. 291 Feb. 2001.
6	Nature, Human Genome Volume, Vol. 409, Feb. 2001.
7	Science, Human Genome Volume, Vol. 291 Feb. 2001.
8	PA Singer, AS Daar (2001). Harnessing Genomics and Biotechnology to Improve Global Health Equity. Science, 294 pp87–89
9	PA Singer, AS Daar (2000). Avoiding Frankendrugs. Nature Biotechnology, 18(12) 1225.
10	Walter W. Powell (1998). "Learning from Collaboration: Knowledge and Networks in the Biotechnology and Pharmaceutical Industries". California Management Review, vol. 40 (3), Spring.
11	Calestous Juma and Norman Clark. "Technological Catch-up: Opportunities and Challenges for Developing Countries". SUPRA Occasional Paper, Research Centre for the Social Sciences, University of Edinburgh (February, 2002).
12	Von Hippel, E. 1986. Lead Users: a source of novel product concepts. Management Science, Vol. 32, No. 7, pp. 791–805.
13	OECD, 1998. National Systems of Innovation. OCED, Paris.
14	1. Stefan Thomke, Ashok Nimgade (2001). "Millenium Pharmaceuticals, Inc." Harvard Business Law Review. 24pp.
15	2. Ray A. Goldberg. "Gene Research, the Mapping of Life and the Global Economy". Harvard Business Review. 58pp.
16	Philippe Cullet. "Trips and the Human Right to Health in Developing Countries". International Environmental Law Research Centre. (See )
17	Jean O. Lanjouw (April 2001)."A Patent Policy Proposal for Global Diseases". Yale University, Brookings Institution and the NBER
18	Hartley & Hartley. "Limitations on using existing legal doctrines in addressing changes in technology: the example of the "Fertility Fraud" cases at UC Irvine". See Hartley & Hartley Attorneys at Law (California) at
19	"Declaration on the TRIPS Agreement and Public Health" (2001). WTO Ministerial Meeting, Doha, Qatar.
20	A.S. Daar, J.-F. Mattei. Appendix 2: Draft Guiding Principles and Recommendations, with alternative suggestions, after receiving comments. Medical Genetics and Biotechnology: Implications for Public Health. December 1999, World Health Organization.
21	A.S. Daar, J.-F. Mattei. Chapter 6: The Human Genome Diversity Project. Medical Genetics and Biotechnology: Implications for Public Health. December 1999, World Health Organization.
22	A.S. Daar, J.-F. Mattei. Chapter 7: Issues Raised by Conducting Research With Indigenous and Genetically Defined Communities. Medical Genetics and Biotechnology: Implications for Public Health. December 1999, World Health Organization.
23	HUGO Ethics Committee. Statement on Benefit-Sharing. April 9, 2000.
24	B.M. Knoppers, M. Hirtle, S. Lormeau. Statement on the Principled Conduct of Genetic Research. HUGO Ethical, Legal, and Social Issues Committee Report to HUGO Council, March 1996.
25	Statement of the WHO Expert Consultation on New Developments in Human Genetics. World Health Organization, 2000.
26	PA Singer, DK Martin, M Giacomini, L Purdy (2000). Priority setting for new technologies in medicine: qualitative case study. BMJ, 321(7272):1316-8.
27	N Daniels (2000). Accountability for reasonableness. BMJ, 321(7272):1300-1.
28	DK Martin, JL Pater, PA Singer (2001). Priority-setting decisions for new cancer drugs: a qualitative case study. Lancet, 358(9294):1676-81.
29	Mugabe, J. et. al. 1996. Managing Access to Genetic Resources: Strategies for Sharing Benefits. ACTS Press, Nairobi.
30	Mugabe, J. and Clark, N. 1997. Technology Transfer and the Convention on Biological Diversity. ACTS Press, Nairobi.
31	Sanchez, V. and Juma, C. 1993. Biodiplomacy. (Chapter 1). ACTS Press, Nairobi.
